# Long Time No Hear, Magnificent *Wohlfahrtia*! Morphological and Molecular Evidence of Almost Forgotten Flesh Fly in Serbia and Western Balkans

**DOI:** 10.3390/microorganisms12020233

**Published:** 2024-01-23

**Authors:** Stanislav Simin, Snežana Tomanović, Ratko Sukara, Marijana Stefanov, Milan Savović, Bojan Gajić, Vesna Lalošević

**Affiliations:** 1 Faculty of Agriculture, Department of Veterinary Medicine, University of Novi Sad, Trg Dositeja Obradovića 8, 21000 Novi Sad, Serbia; marijana.stefanov96@gmail.com (M.S.); lvesna@polj.uns.ac.rs (V.L.); 2 Group for Medical Entomology, Centre of Excellence for Food- and Vector-Borne Zoonoses, Institute for Medical Research—National Institute of Republic of Serbia, University of Belgrade, 11129 Belgrade, Serbia; snezanat@imi.bg.ac.rs (S.T.); ratko.sukara@imi.bg.ac.rs (R.S.); 3 Private Veterinary Practice “MSV Medicus”, Milice Stojadinović Srpkinje 1, 21209 Bukovac, Serbia; msvmedicus@gmail.com; 4 Department of Veterinary Medicine, College of Agriculture and Veterinary Medicine, United Arab Emirates University, Al Ain P.O. Box 15551, United Arab Emirates; b.gajic@uaeu.ac.ae

**Keywords:** *Wohlfahrtia magnifica*, flesh fly, traumatic myiasis, wound myiasis, molecular evidence, sheep, Serbia, Western Balkans

## Abstract

The “beautiful viviparous fly”, *Wohlfahrtia magnifica*, may have a magnificent appearance due to its striking morphology; however, it is a potentially deadly agent of obligate traumatic myiasis in humans and animals, with a serious impact on welfare and economics. The fly is found across the Palearctic realm, including the Western Balkan region, with reports from former Yugoslavian countries from the first half of the 20th century. In this paper, a recent case of wohlfahrtiosis recorded in Northern Serbia is evidenced using morphological and molecular techniques. Larvae were collected from two adult sheep with severe hoof myiasis and two young sheep with genital and interdigital myiasis. Morphological identification was performed for adults bred from the infested vulva and third-stage larvae (L_3_) collected from the hoof wounds, supported with barcoding sequences of the COI gene obtained from larval pairs from the hoof wounds of older and the genitalia of younger sheep. *W. magnifica* was identified according to the appearance of male fly terminalia and the morphology of L_3_, which was confirmed after the comparison of representative sequences of the COI gene (deposited in GenBank™ under accession numbers MT027108–MT027114) to those available in GenBank™. This finding represents the first reported case of wohlfahrtiosis in the Western Balkans in 80 years, highlighting the need to re-inform relevant stakeholders to achieve adequate disease control.

## 1. Introduction

*Wohlfahrtia magnifica* (Schiner, 1862) (*Diptera: Sarcophagidae*), one of the major flesh fly species, is an obligate larval parasite causing traumatic myiasis in different warm-blooded vertebrates, including humans [1,2,3,4,5,6,7]. While the appearance of an adult fly is magnificent due to its striking morphology (“beautiful viviparous fly” [1]), larval infestations, on the contrary, often present a terrible appearance, especially in myiasis in children [1,5,8]. A fact that furthermore raises concern from the One Health perspective is that *W. magnifica*, besides its zoonotic nature, is associated with *Wohlfahrtiimonas chitiniclastica*, a rare but emerging Gram-negative bacterium capable of causing both local skin/soft tissue infection and sepsis in animals and humans [9,10,11,12,13,14,15].

The greatest impact of this magnificent flesh fly arises from livestock infestations, both in terms of animal welfare and economic loss [5]. Sheep are frequent host species, and if this very painful myiasis is not treated, various problems can occur, including lethargy, depression, inappetence, blindness, lameness, reproduction problems, condition loss and even death [1,5,16,17]. The flies are most active during the hot summer months [3,18], although the fly season can last from March to November, depending on the climate [7]. The larviposition occurs in natural openings (eyes, ears, mouth, genital orifices), wounds or even intact skin, where rapidly developing larvae feed on tissues and body fluids until the third larval stage (L_3_) emerges for pupation [2,3] during a fly season or after a diapause [18].

*W. magnifica* is found throughout the Palearctic realm, from Mediterranean countries in Europe and North Africa along the Eurasian steppe belt and the Middle East, further into Central and East Asia [2,3,5,7,18,19]. Across Europe, *Wohlfahrtia* infests sheep at different altitudes, from the Plateaus of France to the Pannonian and East European plains, including the continents’ largest peninsulas, the Iberian, Apennine and Balkan, with increasing prevalence towards the east (Romania, Bulgaria, former USSR) [5,7,17,18].

According to the available literature, *W. magnifica* is present in the Western Balkan countries of former Yugoslavia (Serbia, (North) Macedonia and Croatia) and Albania [2,3,20,21,22,23]. A few published studies show records of *W. magnifica* related to museum collections, caught adult flesh flies during entomological research and clinical cases of myiasis in animals and humans. Baranoff [24] listed one male specimen of *W. magnifica* as a part of the entomological collection in the Zoological Museum in Zagreb, Croatia, but originating from Sarepta, Russia. Adult flies from the environment were also found by Baranoff, in Golubac on the Danube, Serbia and in Loznica near Dubrovnik, Croatia [25,26]. The first clinical cases of *W. magnifica* myiasis in the Western Balkans were reported during WWI in (North) Macedonia; amongst findings in horses and pigs, myiasis was frequently found in sheep, in up to 10% of animals during July and August, particularly in the prepuce of rams [27,28]. In 1925 and 1926, two cases of human myiasis caused by *W. magnifica* were described at the Clinic for Skin and Venereal Diseases of the State Hospital in Skoplje, Macedonia [25]. Both patients had a primary disease with maggot-contaminated wounds (a 13-year-old boy who suffered from severe fungal infection of the scalp (*favus capillitii*) and a 56-year-old male with a tertiary stadium of syphilis (Lues III), with ulcerative wounds on the right leg). A year later, in two regions of Macedonia (Skoplje and Ovče Polje), Baranoff and Ježić [29] recorded traumatic myiasis in sheep and six-month-old pigs, with the majority of infested wounds on the head (mouth and eyes), hooves and skin. In the period between the two World Wars, during investigations of endo and ectoparasite fauna in sheep in former Yugoslavia (samples from Slovenia and Croatia), Mikačić reported myiasis caused by *W. magnifica* [30]. It seems that in the following years and during WWII, there was an increase in the number of cases of wohlfahrtiosis of natural orifices and wounds in pigs and sheep in some parts of Croatia [26,31]. According to Babić and others, a number of cases were recorded in 1942 in the Šid district in pigs (10% of all animals) and the Osijek district in pigs and sheep, especially in the genitalia [31]. In the following year, Baranov (Baranoff) described recorded cases of myiasis in pigs in May in Šid and in August and September in Osijek [26].

Since then, no reports have been published for the Western Balkans in eighty years. This paper aims to present sheep traumatic myiasis recorded in Northern Serbia caused by *W. magnifica*, evidenced using morphological and molecular techniques.

## 2. Materials and Methods

### 2.1. Study Area and Examination of Animals

In mid-July 2017, a sheep farm with a previous history of footrot and reported cases of myiasis was visited. The farm was located in the village of Jarkovac (45°16′05″ N; 20°45′18″ E), Central Banat District, Vojvodina Province, Northern Serbia (Supplementary Figure S1 (the location of a sheep farm where cases of traumatic myiasis were recorded)). The climate of Vojvodina is moderate continental, with cold winters and hot, humid summers, with a wide range of extreme temperatures (mean annual surface air temperature is around 11 °C) and a very irregular distribution of precipitation per month (annual amount of precipitation is approximately 606 mm). Surface winds blow from two prevailing opposite directions, the northwest (cold and humid) and the southeast (warm and dry), which contributes to the climate diversity of Vojvodina Province [32]. The flock consisted of 65 grazing mixed breed sheep. At the time of examination, the sheep were resting in the shade around two buildings located at the margin of a large natural pasture. Affected sheep were visually identified, and four individuals were caught, separated from the flock and examined. All animals were safely immobilized, and, before the extraction of larvae, analgesia was provided. Larvae were removed with tweezers and stored separately in labelled plastic containers containing moist cotton until arrival at the laboratory.

The wounds were rinsed with antiseptic and aqueous insecticide solution (organophosphate, diazinon), and a local antibiotic was sprayed. In cases of affected hooves, zinc vitamin cream mixed with copper sulfate was applied and the wounds were bandaged. The animals were treated with injectable macrocyclic lactones (doramectin), systemic antibiotics and additional supportive therapy.

Larvae from each wound were stored in 70% ethanol and preserved for morphological and molecular identification, except for nine larvae from the vulva of infested hogget, which were reared to the adult stage.

### 2.2. Parasitological Techniques

All larvae were counted and their development stage (L_1_–L_3_) was recorded according to the appearance of posterior peritremes [33]. Encountered interinstars were classified in higher stages (e.g., interinstar II–III was counted as L_3_). Morphological identification was performed for 20 randomly selected L_3_ collected from the hoof wounds of the affected sheep and all L_3_ collected in the hoof wound of a hogget. For clearing, larval specimens were kept in 15% potassium hydroxide aqueous solution for 24 h [34] and then dissected to expose the cephalopharyngeal skeleton, anterior and posterior spiracles and thoracic spines as criteria for species determination.

For adult rearing, larvae were first placed in a glass jar filled to a quarter with sterilized substrate and fed with chicken liver until pupation [35]. When all larvae had pupated, a moist cotton ball was added, the remainder of the liver was removed, and sterile gauze was applied over the lid to prevent the escape of newly emerged flies.

The morphological characteristics of the examined larval structures and adult flies were recorded under biological and stereomicroscopes using an iPhone 12 Pro (Apple Inc., Cupertino, CA, USA) through a microscope adapter, LabCam^®^ (iDu Optics^®^, New York, NY, USA), and fly species were identified following keys for larvae [3,33,36,37,38,39,40] and adults [2,3,41,42,43].

### 2.3. Molecular and Phylogenetic Analysis

In addition to the parasitological examination of collected samples, a molecular analysis was performed to confirm the morphological identification of larvae. A small amount of tissue (3 × 3 mm) from each of the eight randomly selected larvae (two from each site, except a hoof wound in a hogget) was taken for the individual isolation of total DNA. Extraction was performed using the Kapa Express Extract Kit (Kapa Biosystems) following a procedure according to the manufacturer. Isolated DNA was kept frozen at −80 °C for later analysis. The universal DNA primers LCO1490 (forward: 5′ GGTCAACAAATCATAAAGATATTGG 3′) and HCO2198 (reverse: 5′ TAAACTTCAGGGTGACCAAAAAATCA 3′), which amplify a 710-bp barcode region of the mitochondrial cytochrome c oxidase subunit I gene (COI), were used for the molecular identification of larval specimens [44]. PCR reactions (total volume of 50 µL) consisted of 17 µL H_2_O, 3 µL of each of the two primers (10 µM), 25 µL of Kapa 2G Robust Hot Start Ready Mix (Kapa Biosystems) and 2 µL of isolated DNA as a template. Cycling conditions were 94 °C for 2 min followed by 35 cycles of 95 °C for 15 s, 53 °C for 15 s and 72 °C for 15 s. The final extension was performed at 72 °C for 10 min. PCR products were subjected to 2% agarose gel electrophoresis and visualized by ethidium bromide staining under ultraviolet light (BioDocAnalyze Darkhood, Biometra, Göttingen, Germany). After confirming successful amplification for all analyzed samples, PCR products were sent for purification and bidirectional sequencing (Macrogen Inc., Macrogen Europe, Amsterdam, The Netherlands). Obtained sequences were aligned using the FinchTV (ver. 1.4.0) and BioEdit (ver. 7.2.5.0) sequence analysis software and then compared to those available in the GenBank™ dataset by Basic Local Alignment Search Tool (BLAST) analysis [45].

The jModeltest 0.1.1 software was used to select the best-fitting substitution model using all eighty-eight proposed models [46]. Based on the best Akaike information criterion score, the best-fitting model for the present dataset was general time-reversible with gamma-distributed rates among sites and proportions of invariant sites (GTR+G+I). Maximum likelihood (ML) was employed to infer the evolutionary relationships of the analyzed sequences, using MEGA X (MEGA X software, version 10.2.6) [47].

## 3. Results

### 3.1. Clinical Findings

Out of the four examined individuals, two were adult female sheep of about two years old, with a hogget and a ram lamb that were six to seven months old. Adult sheep were easily caught as they were reluctant to walk due to the severe lameness caused by the traumatic myiasis of the hooves. Both ewes had a poor body condition and appeared depressed. Prior to examination, the wounds in the feet were contaminated with dirt and were profusely bleeding during inspection, cleaning and larval removal.

On examination of the first ewe (ID number 463640), infestation with flesh fly larvae was recorded on the hind left leg, in and between the claws. The interdigital space and medial claw were most affected, with at least seven separated foci, approximately 6 cm deep, packed with larvae of different stages, mostly L_3_ (Supplementary Figure S2a (Traumatic myiasis of hooves)). The hard horn was completely missing. On the lateral claw, the abaxial part of the hard horn was mostly preserved, including the heel. Free larvae were found crawling under the horn or packed in a wound in the middle of the sole. There was no swelling of the interphalangeal joints. The most severe wounds were recorded on the second ewe (ID number 2505), located on the medial claw of the front right leg, with massive inflammation and swelling of the proximal and distal interphalangeal joints. There was complete destruction of the hard horn, with two 1.5 cm deep wounds on the coronary band, and complete destruction of the sole, with larval clusters located in eight distinctive foci, some of them about 4 centimeters deep (Supplementary Figure S2b (Traumatic myiasis of hooves)). Part of the hard horn of the abaxial wall of the lateral claw was preserved. There were no wounds in the lateral claw at the time of examination; however, signs of severe arthritis were recorded.

In younger animals, genital and interdigital myiasis were recorded. A young ram suffered from an infestation of preputium (Supplementary Figure S3a (Genital myiasis in sheep)). There was marked swelling of the prepuce, with traces of dried blood around the preputial orifice. Larvae were located in two 2–3 cm deep foci and were extracted using tweezers, which instantly caused bleeding. Vulvar and interdigital myiasis were found in a hogget. The vulva was enlarged, edematous and painful to palpation, with partly coagulated bloody discharge present between the labiae. After cleaning the discharge, tightly packed larvae were seen and developed into the L_3_ stage (Supplementary Figure S3b (Genital myiasis in sheep)). Larvae were also found in a single wound located in the interdigital space on the left hind leg of the hogget, without serious destruction of the horn, but with signs of chronic interdigital infection.

During the examination of infested sheep and removal of the larvae, the presence of gravid females of *W. magnifica* seeking a larviposition site in the hoof wounds was recorded. Additionally, many *Lucilia* spp. adults were also visiting the freshly bleeding hoof wound sites. The flies were attracted to the hooves of both examined adult ewes, where the wounds were highly populated with hundreds of larvae.

### 3.2. Morphological Identification of Larvae and Adults

**Figure 1:**
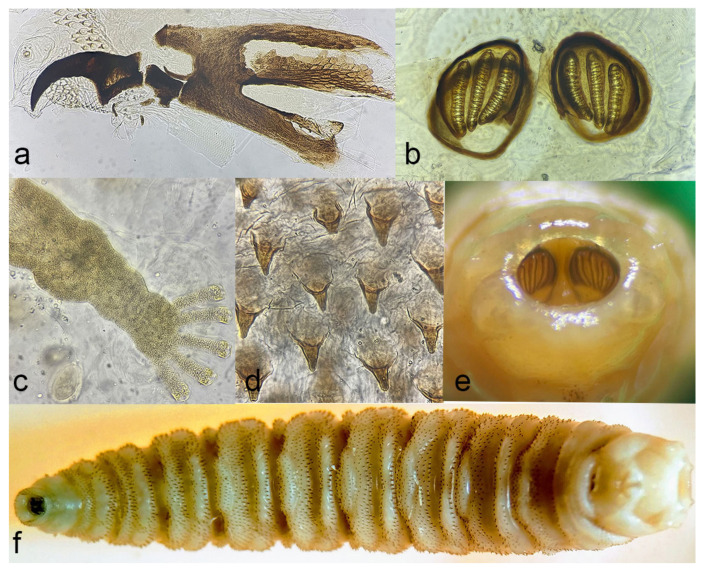
Morphological features of L_3_ of *W. magnifica.* (**a**) Cephalopharyngeal region of L_3_. (**b**) Posterior spiracles. (**c**) Anterior spiracles. (**d**) Thoracic spines. (**e**) Anal plate showing sunken posterior spiracles. (**f**) Ventral presentation of L_3_.

Based on the morphology of the anal division, cephalopharyngeal skeleton, anterior and posterior spiracles and thoracic spines, most L_3_ in hoof wounds were determined as larvae of *W. magnifica* (Figure 1). The co-infestation of a hoof wound with larvae of *Lucilia sericata* was recorded in one animal (ID 4636401) (Table 1).

**Figure 2:**
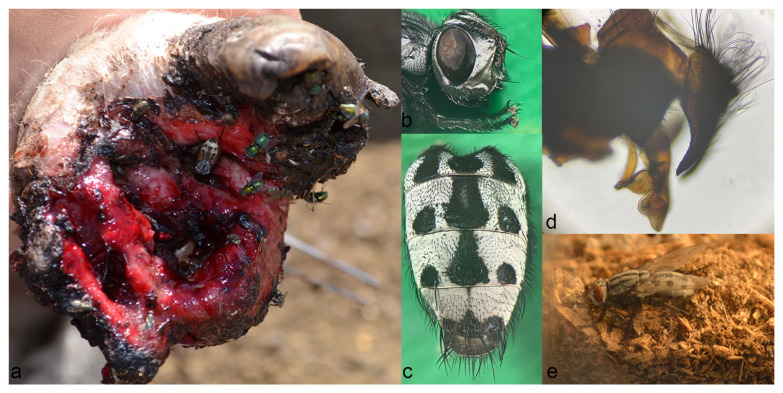
Adults of *W. magnifica.* (**a**) Gravid *W. magnifica* female seeking larviposition site at the hoof wound of already infested sheep. (**b**) Head of adult female with hairless aristae on antennal segments. (**c**) Abdominal pattern of an adult male. (**d**) Male terminalia with the characteristic appearance of the phallus. (**e**) Adult fly reared from vulvar myiasis.

Five flies had emerged fifteen to sixteen days after the pupation of L_3_ larvae extracted from the vulva of an infested hogget. The adult flies (Figure 2) had silver-white shiny parafacial and parafrontal regions of the head, with a forehead as wide as an eye. Several black delicate setulae were recorded at parafacialia. There were eight frontal bristles at each side of the interfrontalia. Antennae were black, with the third antennal segment two times longer than the second. Some adults had a yellowish distal end of the second antennal segment. Aristae thickened at the base, with very short hairs, and then became gradually thinner. The thorax was grey, with three longitudinal stripes. The flies had a characteristic pattern of a grayish abdomen, consisting of a black patch on the first and second tergites shaped like the letter “m”, followed by a median trapezoid black spot with a pair of round black spots each located on the lateral sides on the third to fourth tergite. The median spot of the fifth tergite was triangular, markedly smaller and confined to the rear margin; lateral spots almost merged with a median. The cercus was long, with a curved dorsal outline and slightly pointed. The phallus was sclerotized and curved, with a paddle-shaped apical lobe and a ventral lobe resembling a mastoid process with an elongated blunt tip. According to the appearance of male terminalia, the flies were identified as adults of *W. magnifica*.

### 3.3. Molecular Findings and Phylogenetic Analysis

**Figure 3:**
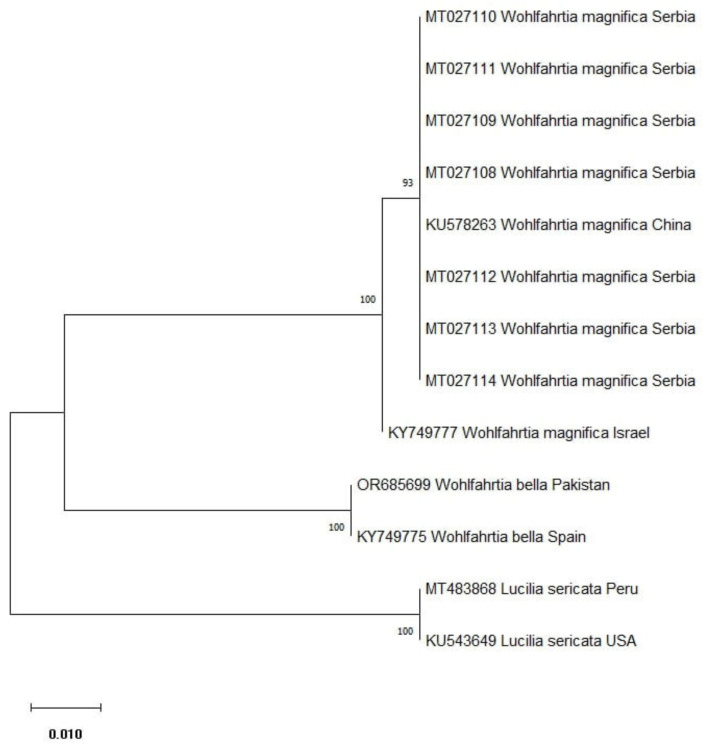
ML phylogenetic tree generated by MEGA X software, version 10.2.6 based on 587 nt of 9 partial cytochrome oxidase subunit I (COI) gene sequences. The numbers in bifurcations indicate bootstrap values (a value of >70 percent was considered as strong nodal support). The tree was rooted with four sequences; two of them were cox1 partial sequences of *Wohlfahrtia bella* (GenBank™ accession numbers: OR685699, KY749775), and two cox1 partial sequences of *Lucilia sericata* (GenBank™ accession numbers: MT483868, KU543649).

Comparing the sequences obtained in this study with those available in GenBank, it was confirmed that all larvae belonged to the *W. magnifica* species, as indicated by the previous morphological identification of larvae and adults. Out of eight sequenced amplicons, seven representative sequences were submitted to GenBank™ under accession numbers MT027108–MT027114 (Table 1), which is the first molecular evidence of *W. magnifica* in Serbia and in the Western Balkans region. Evolutionary analyses by the maximum likelihood method of *W. magnifica* isolates from this study and selected accessions from GenBank™ are presented in Figure 3. Sequences of *W. magnifica* obtained in the present study were clustered together with the sequence of *W. magnifica* from China (KU578263) with high bootstrap support.

## 4. Discussion

The utilization of molecular techniques to support morphological diagnosis provides the final confirmation of *W. magnifica*, as performed in our study, for the first time in the Western Balkans. As previous reports of the magnificent flesh fly in this area date back to the first half of the 20th century, the PCR technique was unavailable. *W. magnifica* in the Western Balkans was mostly identified according to the morphological analysis of larvae extracted from wounds and flies reared from maggots or collected from the environment. Teipel [27] had observed numerous cases of myiasis in surgical and other wounds and natural openings in horses, pigs and sheep, but the attempts to identify flies bred from collected larvae by a zoologist yielded no conclusive results (flies were identified as genus *Sarcophaga*, without more detailed determination). His frequent encounter with genital myiasis (up to 10% of sheep sent for slaughter from Bulgarian warehouses during summer months) could suggest wohlfahrtiosis, as discussed by Hutyra and Marek [28]. The larvae of *W. magnifica* in the wounds of infested sheep were sporadically recorded by Mikačić during post-mortem sections [30]; however, the details regarding these findings were not provided and the rearing and identification of adults were not performed, so Krčmar and others recommended to treat this record with caution [23]. Descriptions of adult *W. magnifica* in the Western Balkans were made by Russian entomologist Nikolay Ilyich Baranoff (Baranov), a specialist in Diptera, who worked in different institutions in Belgrade (Serbia), Skoplje (North Macedonia) and Zagreb (Croatia) [48]. Baranoff collected some specimens in the field during his research of entomofauna in Serbia and Croatia [25,26], but most of them were identified after the rearing of extracted larvae from wound myiasis in cases from North Macedonia, Serbia and Croatia [25,26,29,31]. According to Baranoff, it was necessary to differentiate *W. magnifica* from a very similar species, *W. meigenii*, also present in Croatia [26]. Recently, *W. nuba*, a species atypical for Southern Europe, was reported in Croatia in Split and the Omiš Riviera [49], supporting the need for the accurate differentiation of *Wohlfahrtia* collected in Western Balkan countries. Since the morphological characteristics of adult flies from the genus *Wohlfahrtia* are similar, the proper identification of the species may be challenging. The most reliable means of the morphological identification of adult flies are provided by the examination of male terminalia [42], but misidentification in publications cannot be ruled out, making poorly described records unverified [23].

Historical data regarding the occurrence of *W. magnifica* in Serbia and the Western Balkans may be confusing to readers unfamiliar with the geopolitical timeline of the region. Previously published findings of *W. magnifica* in humans and animals in South Serbia (Südserbien; in German) [25,29] refer to the geographical locations in today’s North Macedonia, which was formerly a part of Serbia from 1913 to 1945. In contrast, the reports of wohlfahrtiosis in pigs in Croatia [26,31] during 1942 and 1943 (then the Independent State of Croatia; 1941–1945) included a part of the occupied territory of Serbia during WWII, such as the Srem region with the town of Šid, where numerous cases were noted. Our finding of *W. magnifica* in traumatic myiasis in sheep from the village of Jarkovac in Northern Serbia is, actually, the fourth record of the species in the country in its current borders, following reports of collected adult flies in Golubac [25] and cases of pig myiasis in Šid [26,31]. Furthermore, our report represents the first published record of *W. magnifica* myiasis in the Western Balkans region after eight decades.

During an extensive literature search for other Western Balkan countries—Albania, Bosnia and Herzegovina and Montenegro—no published records of the fly were found. Moreover, there was a discrepancy between the literature and actual specimen records for Albania. The occurrence of the fly in the country was first listed by Verves [20] in the Catalogue of Palearctic Diptera; however, there is no record of the species in Albania according to the archive of the Public Health Institute in Tirana [50]. There are plenty of unpublished field data regarding the occurrence of genital myiasis in sheep and cattle, provided by veterinarians, farmers or sheep shearers in Serbia, Bosnia and Herzegovina and Montenegro [51]. As myiasis in sheep genitalia was frequently reported in the literature [5,7,16], observed cases of genital myiasis are strongly suggestive of *W. magnifica*, although published evidence is lacking. The moderate to high prevalence of *W. magnifica* in Hungary, Romania, Bulgaria and Greece [5,16,52,53,54], countries bordering Western Balkan states, suggests that, even if unreported, as in Albania, Bosnia and Herzegovina and Montenegro, it is highly unlikely that this flesh fly is absent at similar latitudes and magnitudes, especially in its most frequent host, the sheep.

Following the analysis of reports published for Western Balkan countries, *W. magnifica* is de facto present in North Macedonia [25,27,28,29], Serbia [25,26,31], as this report, and Croatia [26,31]. Additional studies are needed to determine the occurrence and distribution of the magnificent flesh fly in other Western Balkan countries. According to Sotiraki and Hall, the fact that a fly is found in an area does not mean that it will cause myiasis problems there [55]. This may depend on several factors, as, for example, the access to animals (*Wohlfahrtia* rarely strikes indoors), the production system (dairy sheep more infested), the host breed (foreign breeds are more prone to infestation than local ones), predisposing conditions (castration or traumatic wounds, footrot) or the fly strain (possibly more aggressive) [5,7,16,18]. Global warming is an additional concern favoring the distribution of thermophilic flies. There are recent reports regarding the geographical spreading and increased period of activity of *W. magnifica* [7,17]. Therefore, a few historical or even recent single-country records of adult fly presence or myiasis cases in the Western Balkans poorly describe the distribution of wohlfahrtiosis in the region, highlighting the requirement for the development of disease risk distribution maps, based on livestock and fly surveys as part of integrated control programs [55].

There are different current and future possibilities for the treatment and control of wohlfahrtiosis, including the manual removal of larvae; the use of insecticides, macrocyclic lactones, insect growth regulators, essential oils and fly bait strategies; the selection of resistant sheep; and the development of vaccines [5,6,16,18,56,57]. Assuming the awareness of farmers and veterinarians regarding traumatic myiasis, and considering the available drugs in Serbia and Western Balkan countries, as shown in Supplementary Table S1 (list of available veterinary medications for treatment of wohlfahrtiosis in Western Balkan countries), a possible preference for some treatment and control options is discussed. The manual removal of larvae is the least practical method for myiasis control. It is time-consuming and labor-intensive, which is easily evident in large herds and flocks. During the summer season, the animals could be inspected from monthly up to biweekly intervals at the peak of fly activity due to frequent reinfestations [16,56]. Insecticides could offer a better solution against *W. magnifica* infestations. Firstly, organophosphate or pyrethroid insecticides are available in most Western Balkan countries. Since organophosphates have short residual activity [56], pyrethroids could be used since, in other studies, they have provided protection for up to two weeks [4,17,58]. The use of macrocyclic lactones (MLs) for the treatment and prevention of myiasis would probably be one of the first choices of farmers and vets in the region. MLs are endectocides, and they are widely available through local markets, especially ivermectin (IVM), which is also cheap. Regarding myiasis, the use of IVM protected sheep in a range from 12 to 26 days [5,58,59] or was completely ineffective, as with moxidectin [60]. Other MLs, including doramectin and eprinomectin, were protective for 22 and 26 days, respectively [4,5]. Although IVM showed some conflicting results against *W. magnifica*, it is often the first choice of veterinarians, as evidenced in Hungary [56]. Moreover, according to the authors’ experience, most of the farmers and vets would choose a cheaper product, at least in Serbia. There is a serious disadvantage of frequent IVM usage in the therapy and prophylaxis of myiasis, due to widespread anthelmintic resistance in the gastrointestinal nematodes of ruminants, as evidenced recently [61]. The use of very effective insect growth regulators in the prevention of wohlfahrtiosis (dicyclanil achieved 100% of protection in sheep during at least 24 weeks in Crete, Greece [62]) is unfortunately currently unavailable in the Western Balkans as the drug is not registered in any of the countries. Two more promising options to manage *W. magnifica* were published recently. A novel plant-derived formulation, 1PWD©, for wound myiasis treatment was tested in various animals [6]. Differently located wounds, even old ones, successfully healed after 23 days on average. Besides the healing effect, the plant-derived wound dressing had a repellent effect against the reinfestation of larvae. This preparation might find its use in organic animal farming in the Western Balkans region and beyond. Chinese scientists managed to isolate and identify volatile substances with attractive effects on *W. magnifica* from the vaginas of Bactrian camels and laid the foundation for the biological control of traumatic myiasis by using synthetic volatile organic compounds as attractants for odor bait traps [57].

It is of high importance to re-inform animal breeders, veterinarians and physicians about this forgotten flesh fly in our region. Apart from the economic consequences and difficult management, especially in the sheep industry [5,16,56], it is even more important to remember that wohlfahrtiosis causes serious pain for its host [1,17]. It is evident that, especially in cases of neglected infestations, such as the hoof myiasis described in this paper, the animals undergo extensive unnecessary suffering. An example of the pain experienced by all severely infested animals is provided by “wormy Karolina”, a 23-year-old Belorussian girl who suffered from severe recurrent myiasis of the thorax at the end of the 19th century, when treatment options were limited [1]. The cries of this poor girl, fearing death from the maggots, illustrate the necessity of educating the stakeholders in Western Balkan states to achieve the adequate control of wohlfahrtiosis in affected animals, which would also prevent infestation among humans, in support of the One Health concept.

## Figures and Tables

**Table 1:** Summarized results of sheep traumatic myiasis in Jarkovac village, Northern Serbia.

Animal ID	Site of Infection	Number of Extracted Larvae	Morphological ID of Extracted L_3_	GenBank™ Accession Numbers
Adult ewe 2505	Front right hoof	400 (35 L_1_, 38 L_2_, 327 L_3_)	*W. magnifica* (n = 20)	MT027113 MT027114
Adult ewe 4636401	Hind left hoof	354 (25 L_1_, 67 L_2_, 262 L_3_)	*W. magnifica* (n = 17) *L. sericata* (n = 3)	MT027109 MT027110
A hogget	Vulva	17 L_3_	-	MT027108 -
	Hind left hoof	12 (2 L_2_, 10 L_3_)	*W. magnifica* (n = 10)	Not performed
A young ram	Preputium	13 L_3_	-	MT027111 MT027112

## Data Availability

The data that support the findings of this study are available within the main body of the manuscript, the cited references and the Supplementary Materials.

## Supplementary Materials

The following supporting information can be downloaded at: https://www.mdpi.com/article/10.3390/microorganisms12020233/s1, Figure S1: The location of a sheep farm where the cases of traumatic myiasis were recorded; Figure S2: Traumatic myiasis of hooves; Figure S3: Genital myiasis in sheep; Table S1: The list of available veterinary medications for treatment of wohlfahrtiosis in Western Balkan countries.

## References

[1] Portchinsky I.A. (1916). *Wohlfahrtia magnifica*, Schin. and allied Russian species. The biology of this fly and its importance to man and domestic animals. Trans. Bur. Ent..

[2] James M.T. (1947). The Flies That Cause Myiasis in Man.

[3] Zumpt F. (1965). Myiasis in Man and Animals in the Old World. A Textbook for Physicians, Veterinarians and Zoologists.

[4] Sotiraki S., Stefanakis A., Hall M.J.R. (2003). Assessment of cypermethrin and doramectin for controlling wohlfahrtiosis in Crete. Vet. Parasitol..

[5] Sotiraki S., Farkas R., Hall M.J.R. (2010). Fleshflies in the flesh: Epidemiology, population genetics and control of outbreaks of traumatic myiasis in the Mediterranean Basin. Vet. Parasitol..

[6] Carnevali F., Franchini D., Otranto D., Giangaspero A., Di Bello A., Ciccarelli S., Szpila K., Valastro C., van der Esch A.S. (2019). A formulation of neem and hypericum oily extract for the treatment of the wound myiasis by *Wohlfahrtia magnifica* in domestic animals. Parasitol. Res..

[7] Remesar S., Otero J.L., Panadero R., Díez-Baños P., Díaz P., García-Díos D., Martínez-Calabuig N., Morrondo M.P., Alonso F., López C. (2022). Traumatic myiasis by *Wohlfahrtia magnifica* in sheep flocks from Southeastern Spain: Prevalence and risk factors. Med. Vet. Entomol..

[8] Badri A.R., Harbi A.T., Tonnsi A., Almatry A., Hassanein R. (2016). Cutaneous myiasis in a child scalp caused by *Wohlfahrtia magnifica* (Diptera: Sarcophagidae): A case report. MOJ Clin. Med. Case Rep..

[9] Tóth E.M., Schumann P., Borsodi A.K., Kéki Z., Kovács A.L., Márialigeti K. (2008). *Wohlfahrtiimonas chitiniclastica* gen. nov., sp. nov., a new gammaproteobacterium isolated from *Wohlfahrtia magnifica* (Diptera: Sarcophagidae). Int. J. Syst. Evol. Microbiol..

[10] Rebaudet S., Genot S., Renvoise A., Fournier P.E., Stein A. (2009). *Wohlfahrtiimonas chitiniclastica* Bacteremia in Homeless Woman. Emerg. Infect. Dis..

[11] Almuzara M.N., Palombarani S., Tuduri A., Figueroa S., Gianecini A., Sabater L., Ramirez M.S., Vay C.A. (2011). First case of fulminant sepsis due to *Wohlfahrtiimonas chitiniclastica*. J. Clin. Microbiol..

[12] Thaiwong T., Kettler N.M., Lim A., Dirkse H., Kiupel M. (2014). First Report of emerging zoonotic pathogen *Wohlfahrtiimonas chitiniclastica* in the United States. J. Clin. Microbiol..

[13] Qi J., Gao Y., Wang G.S., Li L.B., Li L.L., Zhao X.M., Du Y.J., Liu Y.Q. (2016). Identification of *Wohlfahrtiimonas chitiniclastica* isolated from an infected cow with hoof fetlow, China. Infect. Genet. Evol..

[14] Suraiya S., Zuraina N., Ahmad F., Rahman Z.A. (2017). Fatal *Wohlfahrtiimonas chitiniclastica* bacteremia in an immunocompromised patient. Clin. Microbiol. Newsl..

[15] Hladik M., Lipovy B., Kaloudova Y., Hanslianova M., Vitkova I., Deissova T., Kempny T., Svoboda M., Kala Z., Brychta P. (2021). Human infections by *Wohlfahrtiimonas chitiniclastica*: A Mini-Review and the First Report of a Burn Wound Infection after Accidental Myiasis in Central Europe. Microorganisms.

[16] Farkas R., Hall M.J.R., Kelemen F. (1997). Wound myiasis of sheep in Hungary. Vet. Parasitol..

[17] Jacquiet P.P., Alzieu J.P., Liénard E., Grisez C., Prévot F., Bergeaud J.P., Bouhsira E., Franc M., Dorchies P. (2016). Évolutions epidémiologiques et nouvelles contraintes dans a lutte contre les myiases ovines. Bull. Acad. Vet. Fr..

[18] Hall M.J.R. (1997). Traumatic myiasis of sheep in Europe: A review. Parassitologia.

[19] Li Y., Li X., Liu J., Liu A., Guo P., Han Y., Shang Y., Guan G., Liu Z., Liu G. (2019). Molecular identification and detection of *Wohlfahrtia magnifica* in ovine vulvar myiasis in Gansu, China. Trop. Anim. Health Prod..

[20] Verves Y.G., Soós Á., Papp L. (1986). Family *Sarcophagidae*. Catalogue of Palaearctic Diptera.

[21] Pape T. (1996). Catalogue of the *Sarcophagidae* of the world (*Insecta: Diptera*). Mem. Ent. Int..

[22] Verves Y.G., Khrokalo L.A. (2014). An annotated list of the *Sarcophagidae* (*Macronychiinae*, *Miltogramminae*, *Eumacronychiinae* and *Paramacronychiinae*) recorded in Ukraine (*Diptera*). CESA News.

[23] Krčmar S., Whitmore D., Pape T., Buenaventura E. (2019). Checklist of the Sarcophagidae (Diptera) of Croatia, with new records from Croatia and other Mediterranean countries. Zookeys.

[24] Baranoff N. (1928). Tachinidensammlung des zoologischen Museums in Zagreb. Glas. Hrvat. Prir. Društva.

[25] Kislitschenko L., Baranoff N. (1927). Fliegenmaden als Wundenschmarotzer in Süd-Serbien (Mazedonien). Dermatol. Wochenschr..

[26] Baranov N. (1943). *Wohlfahrtia magnifica* Schin., als Erreger der Schweine-Myiasis in Kroatien. Vet. Arh..

[27] Teipel H. (1918). Bösartige Einwirkungen von Fliegenlarven. Ztschr. F. Veterinärk..

[28] Hutyra F., Marek J. (1922). Spezielle Pathologie und Therapie der Haustiere, Dritter Band.

[29] Baranoff N., Ježić J. (1928). Fliegenmaden als Wundschmarotzer bei den Haustieren in Südserbien. Z. Parasitenkd..

[30] Mikačić D. (1938). La faune parasitaire des moutons en Yougoslavie. Vet. Arh..

[31] Babić I., Mikačić D., Šlezić M. (1943). Parasites and Parasitic Diseases of Pigs.

[32] Gavrilov M.B., Radaković M.G., Sipos G., Mezősi G., Gavrilov G., Lukić T., Basarin B., Benyhe B., Fiala K., Kozák P. (2020). Aridity in the Central and Southern Pannonian Basin. Atmosphere.

[33] Ruiz-Martinez I., Soler-Cruz M.D., Benitez-Rodriguez R., Perez-Jimenez J.M., Diaz-Lopez M. (1989). Postembryonic development of *Wohlfahrtia magnifica* (Schiner, 1862) (*Diptera: Sarcophagidae*). J. Parasitol..

[34] Nigoghosian G., Weidner L.M., Stamper T.I. (2021). A technique to mount *Sarcophagidae* and *Calliphoridae* (*Diptera*) larvae for forensic identification using geometric morphometrics. Forensic Sci. Int. Synerg..

[35] Bowman D.D. (2009). Georgis’ Parasitology for Veterinarians.

[36] Kokcam I., Saki C.E. (2005). A case of cutaneous myiasis caused by *Wohlfahrtia magnifica*. J. Dermatol..

[37] Szpila K., Amendt J., Goff M., Campobasso C., Grassberger M. (2010). Key for the Identification of Third Instars of European Blowflies (*Diptera: Calliphoridae*) of Forensic Importance. Current Concepts in Forensic Entomology.

[38] Fremdt H., Szpila K., Huijbregts J., Lindström A., Zehner R., Amendt J. (2012). *Lucilia silvarum* Meigen, 1826 (*Diptera: Calliphoridae*)—A new species of interest for forensic entomology in Europe. Forensic Sci. Int..

[39] Szpila K., Richet R., Pape T. (2015). Third instar larvae of flesh flies (*Diptera: Sarcophagidae*) of forensic importance—Critical review of characters and key for European species. Parasitol. Res..

[40] An X., Yang B., Bao H., Oyun G., Wang X., Er D. (2019). Morphological observation of the larva of the alxa bactrian camel vaginal myiasis. J. Camel Pract. Res..

[41] Rohdendorf B.B. (1956). The Palaearctic species of the genus *Wohlfahrtia* B.B. (*Diptera, Sarcophagidae*). Ent. Obozr..

[42] Ge Y.Q., Zhang D., Pape T. (2018). A new species of *Wohlfahrtia* Brauer & Bergenstamm (*Diptera: Sarcophagidae*) from northwestern China, with three new synonymies and a pictorial synopsis. Zootaxa.

[43] Li H., An X., Zhou J., Ba L., Cha H., Bao H., Yang B., Li Y., Er D. (2020). Morphological observation of the *Wohlfahrtia magnifica* in mongolia plateau. J. Camel Pract. Res..

[44] Folmer O., Black M., Hoeh W., Lutz R., Vrijenhoek R. (1994). DNA primers for amplification of mitochondrial cytochrome c oxidase subunit I from diverse metazoan invertebrates. Mol. Mar. Biol. Biotechnol..

[45] BLAST: Basic Local Alignment Search Tool. http://blast.ncbi.nlm.nih.gov/Blast.cgi.

[46] Posada D. (2008). JModelTest: Phylogenetic Model Averaging. Mol. Biol. Evol..

[47] Kumar S., Stecher G., Li M., Knyaz C., Tamura K. (2018). MEGA X: Molecular Evolutionary Genetics Analysis across Computing Platforms. Mol. Biol. Evol..

[48] Sisojević P. (1982). *In memoriam* Nikolaj Iljič Baranov, 1887–1981. Acta Entomol. Jugosl..

[49] Kovačević A. (2021). Comparison of Abundance and Diversity of Carnivorous Fly Species in the Area of the Cities of Split and Omiš. Master’s Thesis.

[50] Bizgha B. (2023). Personal communication.

[51] Simin S. (2023). Unpublished work.

[52] Nedelchev N.K. (1988). Distribution and causes of myiasis among farm animals. Vet. Sb..

[53] Lehrer A.Z., Lehrer M., Verstraeten C. (1988). Les myiases causées aux moutons de Roumanie par *Wohlfahrtia magnifica* (Schiner) (*Diptera: Sarcophagidae*). Ann. Med. Vet..

[54] Mot D. (2013). The prevalence of sheep traumatic myiasis in Western Romania and bacteria Isolated from the insects maggots. Sci. Pap. Anim. Sci. Biotechnol..

[55] Sotiraki S., Hall M.J.R. (2012). A review of comparative aspects of myiasis in goats and sheep in Europe. Small Rumin. Res..

[56] Farkas R., Hall M.J.R. (1998). Prevalence of traumatic myiasis in Hungary: A questionnaire survey of veterinarians. Vet. Rec..

[57] Xue J., Ai D., Xu X., Wang C., Jiang X., Han T., Er D. (2022). Isolation and Identification of Volatile Substances with Attractive Effects on *Wohlfahrtia magnifica* from Vagina of Bactrian Camel. Vet. Sci..

[58] Al-Eissa G.S., Gammaz H.A., Mohamed Hassan M.F., Abdel-Fattah A.M., Al-Kholany K.M., Halamy M.Y. (2008). Evaluation of the therapeutic and protective effects of ivermectin and permethrin in controlling of wound myiasis infestation in sheep. Parasitol. Res..

[59] Ruiz-Martínez I. (1995). The efficacy of ivermectin against the screwworm fly, *Wohlfahrtia magnifica* (Schiner 1862). Res. Rev. Parasitol..

[60] Farkas R., Hall M.J.R., Daniel M., Börzsönyi L. (1996). Efficacy of ivermectin and moxidectin injection against larvae of *Wohlfahrtia magnifica* (Diptera: Sarcophagidae) in sheep. Parasitol. Res..

[61] Rose Vineer H., Morgan E.R., Hertzberg H., Bartley D.J., Bosco A., Charlier J., Chartier C., Claerebout E., de Waal T., Hendrickx G. (2020). Increasing importance of anthelmintic resistance in European livestock: Creation and meta-analysis of an open database. Parasite.

[62] Sotiraki S., Stefanakis A., Hall M.J.R., Graf J.F. (2005). Field Trial of the efficacy of dicyclanil for the prevention of wohlfahrtiosis of sheep. Vet. Rec..

