# Supplementary material for: Long Time No Hear, Magnificent Wohlfahrtia! Morphological and Molecular Evidence of Almost Forgotten Flesh Fly in Serbia and Western Balkans
Source: Microorganisms. 2024 Jan 23;12(2):233. doi: 10.3390/microorganisms12020233 (PMC10893005; doi:10.3390/microorganisms12020233)
Supplement: Supplementary file 1 [file microorganisms-12-00233-s001.zip › Figure S3.pdf]

Supplementary material – Figure S3

Long time no hear, magnificent *Wohlfahrtia*! Morphological and molecular evidence of almost forgotten flesh fly in Serbia and Western Balkans

Stanislav Simin[1,*], Snežana Tomanović[2], Ratko Sukara[2], Marijana Stefanov[1], Milan Savović[3], Bojan Gajić[4] and Vesna Lalošević[1]

[1]University of Novi Sad, Faculty of Agriculture, Department of Veterinary Medicine, Trg Dositeja Obradovića 8, 21000 Novi Sad, Serbia; stanislav.simin@polj.edu.rs, marijana.stefanov96@gmail.com, lvesna@polj.uns.ac.rs

[2]University of Belgrade, Institute for Medical Research- National Institute of Republic of Serbia, Group for Medical Entomology, Centre of Excellence for Food- and Vector-Borne Zoonoses, 11129 Belgrade, Serbia; snezanat@imi.bg.ac.rs, ratko.sukara@imi.bg.ac.rs

[3]Private Veterinary Practice „MSV Medicus", Milice Stojadinović Srpkinje 1, 21209 Bukovac, Serbia; msvmedicus@gmail.com

[4]United Arab Emirates University, College of Agriculture and Veterinary Medicine, Department of Veterinary Medicine, P.O. Box 15551, Al Ain, United Arab Emirates; b.gajic@uaeu.ac.ae

*Correspondence: stanislav.simin@polj.edu.rs

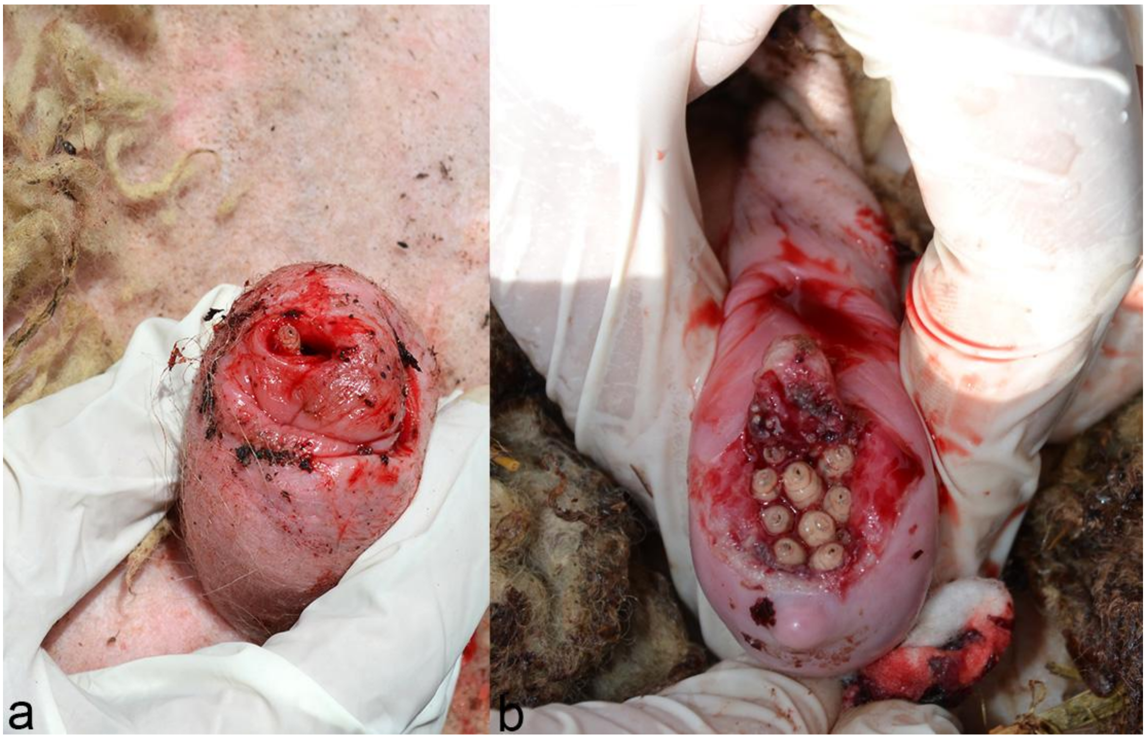

Figure S3. Genital myiasis in sheep. a) prepuce of a young ram; b) vulva of a hogget.
