# Supplementary material for: Long Time No Hear, Magnificent Wohlfahrtia! Morphological and Molecular Evidence of Almost Forgotten Flesh Fly in Serbia and Western Balkans
Source: Microorganisms. 2024 Jan 23;12(2):233. doi: 10.3390/microorganisms12020233 (PMC10893005; doi:10.3390/microorganisms12020233)
Supplement: Supplementary file 1 [file microorganisms-12-00233-s001.zip › Table S1.pdf]

Supplementary material – Table S1

Long time no hear, magnificent *Wohlfahrtia*! Morphological and molecular evidence of almost forgotten flesh fly in Serbia and Western Balkans

Stanislav Simin[1,*], Snežana Tomanović[2], Ratko Sukara[2], Marijana Stefanov[1], Milan Savović[3], Bojan Gajić[4] and Vesna Lalošević[1]

[1]University of Novi Sad, Faculty of Agriculture, Department of Veterinary Medicine, Trg Dositeja Obradovića 8, 21000 Novi Sad, Serbia; stanislav.simin@polj.edu.rs, marijana.stefanov96@gmail.com, lvesna@polj.uns.ac.rs
[2]University of Belgrade, Institute for Medical Research- National Institute of Republic of Serbia, Group for Medical Entomology, Centre of Excellence for Food- and Vector-Borne Zoonoses, 11129 Belgrade, Serbia; snezanat@imi.bg.ac.rs, ratko.sukara@imi.bg.ac.rs
[3]Private Veterinary Practice „MSV Medicus", Milice Stojadinović Srpkinje 1, 21209 Bukovac, Serbia; msvmedicus@gmail.com
[4]United Arab Emirates University, College of Agriculture and Veterinary Medicine, Department of Veterinary Medicine, P.O. Box 15551, Al Ain, United Arab Emirates; b.gajic@uaeu.ac.ae
*Correspondence: stanislav.simin@polj.edu.rs

Table S1. The list of available veterinary medications for treatment of wohlfahrtiosis in Western Balkan countries.

Country	Macrocyclic lactones	Organophosphates	Insect growth regulators	Pyrethroids
Albania[a]	Abamectin, Ivermectin	Diazinon, Phoxim	/	Cypermethrin, Deltamethrin
Bosnia and Herzegovina[b]	Ivermectin	Diazinon	/	/
Croatia[c]	Abamectin, Doramectin, Eprinomectin, Ivermectin, Moxidectin	Diazinon, Phoxim	/	Deltamethrin
Montenegro[d]	Ivermectin	Diazinon	/	/
North Macedonia[e]	Abamectin, Ivermectin	Diazinon, Phoxim	/	Cypermethrin, Deltamethrin
Serbia[f]	Doramectin, Eprinomectin, Ivermectin	Diazinon, Phoxim	/	Deltamethrin

[a] https://bujqesia.gov.al/lista-e-produkteve-mjekesore-veterinare-te-regjistruara-ne-republiken-e-shqiperise/
[b]https://www.vet.gov.ba/bs/registri/c241
[c] http://www.veterinarstvo.hr/default.aspx?id=140
[d] https://cinmed.me/registar-veterinarskih-ljekova/#anchor-id
[e]https://fva.gov.mk/mk/registri-veterinarno-medicinski-preparati
[f]https://www.alims.gov.rs/english/veterinary-medicines/search-for-veterinary-medicines/
All web sites were accessed at 11th of November 2023.
